# Radon balneotherapy in rheumatologic diseases: potential mechanisms and implications for cardiovascular autonomic control and inflammation

**DOI:** 10.1007/s10067-026-08202-y

**Published:** 2026-06-24

**Authors:** Gabriel D. Rodrigues, Earric Lee, Benoit Dugué, Christian Hanshans, Maria Chiara Maccarone, Konstantinos Triantafyllias, Arnulf Hartl, Eugenijus Kaniusas, Sabrina Mörkl, Thomas E. Schmid, Goda-Camille Mickeviciute, Hannes Untner, Werner Klingler, Martin Offenbächer

**Affiliations:** 1 Department of Physiology and Pharmacology, Federal Fluminense University, Niteroi, Rio de Janeiro, Brazil; 2 Montreal Heart Institute, Montréal, QC Canada; 3 École de Kinésiologie Et Des Sciences de L’activité Physique, Université de Montréal, Montréal, QC Canada; 4 University of Poitiers, Laboratory “Mobilité, Vieillissement, Exercice (MOVE)” - UR 20296, Faculty of Sports Sciences, Poitiers, France; 5 Department of Applied Sciences and Mechatronics, University of Applied Science Munich, 80335 Munich, Germany; 6 Department of Neuroscience (DNS), Section of Rehabilitation, University of Padova, Padua, Italy; 7 Department of Internal Medicine I, Division of Rheumatology and Clinical Immunology, University Medical Center, Johannes Gutenberg University, 55131 Mainz, Germany; 8 Institute of Ecomedicine, Paracelsus Medical University Salzburg, Strubergasse 21, 5020 Salzburg, Austria; 9 Institute of Biomedical Electronics, Vienna University of Technology (TU Wien), Vienna, Austria; 10 Department of Medical Psychology, Psychosomatics and Psychotherapy, Medical University of Graz, Graz, Austria; 11 Department Radiooncology, TUM School of Medicine and Health, TUM University Hospital, Munich, Germany; 12 Center of Rehabilitation, Physical and Sport Medicine, Vilnius University Hospital Santaros Klinikos, 08661 Vilnius, Lithuania; 13 Gastein Healing Gallery, Bad Gastein, Austria; 14 Technische Universität München, Konservative Und Rehabilitative Orthopädie, Munich, Germany; 15 Klinikum Bad Gastein, Bad Gastein, Austria

**Keywords:** Functional capacity, Inflammatory cytokines, Oxidative stress, Pain reduction, Radon baths, Rheumatologic diseases

## Abstract

Radon balneotherapy, a form of low-dose radiation treatment, has been utilized for decades in managing rheumatologic diseases, particularly in Central Europe and parts of Asia. Despite ongoing debate about its efficacy, developing research suggests that low-dose exposure to radon may exert anti-inflammatory and analgesic effects, with potential implications for musculoskeletal and cardiovascular health. This narrative review aims to synthesize current knowledge on the biological mechanisms underlying radon therapy, evaluate clinical evidence for its efficacy in rheumatologic conditions, and explore its potential impact on cardiovascular health. Risks, controversies, and regulatory restrictions are also addressed. Preclinical studies indicate that radon therapy modulates oxidative stress responses, immune signalling cascades, and pro-inflammatory cytokine profiles, thereby influencing tissue homeostasis and pain perception. Clinically, benefits have been observed in patients with rheumatoid arthritis, ankylosing spondylitis, and osteoarthritis, particularly with respect to pain alleviation, functional improvement, and quality of life. However, heterogeneity in treatment protocols, study quality, and confounding factors such as the use of heat, carbon dioxide, and/or other concurrent modalities limit generalizability and attributability. Evidence regarding cardiovascular safety remains sparse but suggests that low-dose exposure may have neutral or even protective effects under specific physiological conditions. In summary, radon balneotherapy may have a complementary role alongside other modalities like heat and negative air ions. However, the isolated therapeutic efficacy, long-term safety profile, and cardiovascular implications of radon balneotherapy remain insufficiently characterized. Higher quality multi-armed randomized controlled trials and isolated mechanistic investigations are needed to better delineate its biological mechanisms, optimize protocols, and define its clinical niche.

**Supplementary Information:**

The online version contains supplementary material available at 10.1007/s10067-026-08202-y.

## Introduction

Rheumatologic diseases encompass a broad spectrum of chronic, often progressive diseases that affect the joints, connective tissues, and the immune system [1, 2]. Conditions such as rheumatoid arthritis, ankylosing spondylitis, and osteoarthritis involve not only local musculoskeletal damage but also systemic effects extending beyond the joints [3–7]. These include increased cardiovascular risk [3], fatigue [4], autonomic nervous system (ANS) dysregulation [5, 6], and overall reduced quality of life [7]. A triad of key pathophysiological processes underpins many rheumatologic conditions: persistent inflammation, chronic pain, and disturbances in the autonomic function, all of which contribute to disease burden and psychiatric comorbidities [8].

In recent years, interest has grown in complementary and integrative treatment approaches aimed at counterbalancing these systemic processes [9, 10]. One such approach is radon balneotherapy, a traditional yet still widely practiced intervention in various European spa regions [11, 12]. The use of natural health resources in the treatment of chronic and inflammatory diseases has a long-standing tradition, particularly in regions rich in geothermal and mineral springs [13, 14]. Among these, radon balneotherapy occupies a unique place in medical history. Originating in Central Europe and practised for centuries in countries such as Austria, Germany, and the Czech Republic, as well as in Japan, it combines therapeutic exposure to low-dose radon gas with immersion in mineral-rich waters [15, 16]. Despite initial scepticism, especially in contemporary scientific discourse, the sustained clinical application and body of evidence supporting its effects for conditions such as rheumatoid arthritis and ankylosing spondylitis have renewed interest in this integrative approach [17]. The historical and empirical foundation of radon balneotherapy provides an important backdrop for exploring its physiological mechanisms and modern clinical relevance.

Radon balneotherapy involves exposure to low doses of naturally occurring radon gas through immersion in mineral-rich thermal waters, often accompanied by serial heat applications (e.g., heat therapy) and the inhalation of negative air ions (NAIs) generated by radon decay. These combined environmental stimuli are believed to act synergistically, producing systemic effects through redox modulation, immune regulation, and ANS balance [18, 19]. From a radiobiological perspective, low-dose alpha irradiation may activate adaptive cellular defence pathways, such as enhanced antioxidant enzyme activity, DNA repair mechanisms, and anti-inflammatory signalling, while mild heat and ionic stimuli further potentiate these effects via improved circulation and neuroimmune modulation. The growing recognition of the bidirectional brain–immune axis and the influence of environmental inputs on neuroimmune homeostasis appears to corroborate these findings.

The aim of this narrative review is (1) to synthesize current clinical evidence on the use of radon balneotherapy in managing these conditions; (2) to outline the role of the ANS in the pathophysiology of rheumatologic diseases; (3) to explore the radiobiological mechanisms through which low-dose radon exposure, therapeutic hyperthermia, and negative air ions (NAIs) may exert anti-inflammatory, antioxidative, and autonomic-regulatory effects; (4) to assess the potential of these environmental interventions to modulate cardiovascular risk and vascular homeostasis via ANS-mediated pathways, in comparison with other non-pharmacological or spa-based therapies; and (5) to discuss the associated risks, scientific controversies, and current regulatory constraints surrounding radon use in medicine. The review aims to highlight radon therapy as a systemic treatment modality, rather than merely a localized or symptomatic intervention, within the broader context of radiobiological adaptation and low-dose radiation hormesis.

## Methods

This review was conducted as a narrative synthesis rather than a systematic review. This design choice was made for three specific reasons: (1) the exploratory aim of integrating disparate literature streams (clinical rheumatology, low-dose radiobiology, autonomic physiology, and balneology) which span fundamentally different study designs and evidentiary standards not amenable to pooled meta-analysis; (2) the heterogeneity of radon therapy protocols (varying exposure durations, concentrations, and co-interventions) which precludes meaningful quantitative synthesis; and (3) the intended purpose of this review as a hypothesis-generating framework to identify research gaps rather than to produce clinical practice recommendations.

In accordance with the Scale for the Assessment of Narrative Review Articles (SANRA) [20], the following sections transparently report the literature search strategy, selection process, and synthesis approach.

### Literature search strategy

To identify potential articles, a comprehensive literature search was performed using the electronic databases PubMed, Scopus, and Web of Science from their inception until July 2025. The search strategy utilized a combination of keywords and Medical Subject Headings (MeSH) terms related to the intervention and outcomes. Search terms reflected the core concepts of the review: radon balneotherapy in rheumatologic diseases, specifically cardiovascular autonomic control and inflammation. Key search terms thus included: (“radon bath” OR “radon therapy” OR “radon balneotherapy” OR “low-dose radon”) AND (“rheumat” OR “arthritis” OR “ankylosing spondylitis” OR “osteoarthritis” OR “fibromyalgia”) AND (“autonomic nervous system” OR “heart rate variability” OR “vagal tone” OR “sympathetic”) AND (“inflammation” OR “cytokine” OR “oxidative stress”). The reference lists of retrieved articles were also manually screened for additional relevant publications.

### Study selection and inclusion criteria

Articles were considered eligible if they addressed one or more of the following domains relevant to the review’s aims: (i) therapeutic or physiological effects of radon exposure; (ii) autonomic nervous system modulation by environmental or balneological interventions; or (iii) anti-inflammatory or oxidative stress-mediating mechanisms in rheumatologic disease.

The following explicit inclusion and exclusion criteria were applied:

Inclusion criteria: (a) Peer-reviewed original research (randomized controlled trials, observational studies, mechanistic investigations in humans or animal models); (b) Systematic reviews and meta-analyses; (c) Studies reporting quantifiable dosimetric parameters or biological endpoints (e.g., cytokine profiles, reactive oxygen species levels, DNA repair markers). Exclusion criteria: (a) Studies exclusively addressing high-dose occupational or mining-related radon exposure without therapeutic context; (b) Non-peer-reviewed sources (conference abstracts, grey literature); (c) Articles for which full-text access could not be obtained.

No restrictions were placed on publication date. However, the authors acknowledge that the selection process was not blinded or independently dual-screened, which introduces potential for selection bias. This limitation is explicitly addressed in the “Limitations” section.

### Data synthesis

Due to substantial heterogeneity in study designs, populations, intervention protocols, and outcome measures, a narrative synthesis approach was adopted. The synthesis was organized thematically according to the pre-specified aims of the review (see the “Introduction” section). When interpreting findings, particular attention was paid to distinguishing between (a) evidence specifically attributable to radon exposure, (b) evidence attributable to the multimodal spa environment (heat, immersion, rehabilitation), and (c) evidence from related but distinct interventions (e.g., negative air ions, forest bathing) that may inform mechanistic hypotheses but should not be conflated with radon-specific effects.

The quality of individual studies was not formally scored using a standardized instrument (e.g., Cochrane Risk of Bias tool), consistent with the narrative design. However, key methodological limitations of the evidence base are critically discussed in the “Limitations” section and are explicitly noted in the accompanying Evidence Summary Table (Supplemental Table 1). The goal of this synthesis is to provide a comprehensive, transparent overview of the current state of knowledge and to identify critical gaps requiring future investigation.

## Autonomic nervous system and rheumatologic diseases

The ANS is modulated by a wide spectrum of intrinsic and extrinsic factors, including metabolic state, psychological stress, inflammation, and environmental stimuli. Heart rate variability (HRV) serves as a robust, non-invasive biomarker of ANS function, allowing for the assessment of cardiac autonomic modulation across chronic disease states and in response to behavioural and environmental interventions [21, 22]. The ANS reflexively regulates innate and adaptive immunity through its sympathetic and parasympathetic branches [6]. An imbalance in this system can lead to an altered inflammatory response [6, 23], as typically observed in rheumatologic diseases such as rheumatoid arthritis, ankylosing spondylitis, osteoarthritis (OA), systemic sclerosis, and fibromyalgia (FM). These conditions exhibit ANS dysfunction, which is associated with sympathetic dominance, pro-inflammatory status and an increased cardiovascular risk [24, 25]. In this context, interventions capable of restoring autonomic balance, such as low-dose radon therapy, heat therapy, or other environmental modalities, may offer cardiometabolic and anti-inflammatory benefits via activation of the parasympathetic (cholinergic anti-inflammatory) pathway.

### Rheumatoid arthritis

In rheumatoid arthritis patients, resting peripheral muscle sympathetic activity and heart rate positively correlate with pro-inflammatory cytokines. Additionally, higher pain perception is associated with increased resting sympathetic activity [26]. Conversely, cardiac vagal modulation and baroreflex sensitivity show inverse correlations with multiple pro-inflammatory cytokines, such as IFN-γ, IL-8, MCP-1 and TNFα [26, 27].

### Ankylosing spondylitis

Ankylosing spondylitis is an inflammatory rheumatic disease that affects the spine and sacroiliac joints. Typically, it presents with suppressed cardiac parasympathetic modulation, a reduced baroreflex slope, and an elevated heart rate [28, 29]. Notably, reduced heart rate recovery and vagal-related HRV indices are observed even in asymptomatic ankylosing spondylitis patients, reinforcing their relevance in cardiovascular risk prediction [30, 31]. Moreover, reduced cardiac vagal activity correlates positively with disease activity, inflammatory markers, and structural progression, suggesting that subclinical parasympathetic dysfunction may both reflect and reinforce chronic low-grade inflammation [32]. However, the generalizability of these findings is limited by the scarcity of studies that include direct comparisons with healthy control groups. Similarly, in systemic sclerosis, an autoimmune disorder characterized by vascular damage, autoantibody production, and fibrosis, the sympatho-vagal balance shifts toward sympathetic dominance with vagal withdrawal (at rest) alongside a blunted autonomic response to orthostatic stress when compared to age-matched healthy control [33]. Autonomic dysfunction progresses alongside fibrovascular disease, as confirmed in a follow-up study linking worsening cardiac autonomic impairment to the diffuse scleroderma subset, which presents more extensive skin and organ fibrosis [34]. Moreover, systemic sclerosis patients with elevated pulmonary arterial pressure exhibit a heightened inflammatory state and impaired cardiac autonomic response to orthostatic stress, underscoring the interplay between autonomic regulation and inflammation resulting from the disease [35]. Together, these findings highlight a consistent pattern of autonomic imbalance across inflammatory rheumatic diseases, characterized by parasympathetic withdrawal and sympathetic overactivation, that contributes to endothelial dysfunction, inflammation, and increased cardiovascular morbidity.

### Osteoarthritis

Recent studies have examined the role of the ANS in joint homeostasis and OA progression [36, 37]. Osteoarticular cells, including chondrocytes, osteoblasts, and synoviocytes, express both adrenergic and cholinergic receptors, enabling them to directly respond to autonomic neurotransmitters. Additionally, some of these cells can locally produce and release neurotransmitters, acting independently of autonomic innervation [37]. As in other chronic inflammatory diseases, sympathetic overactivity persists in OA, promoting catabolic and pro-inflammatory signaling within joint tissues [36, 38]. Conversely, the parasympathetic (vagal) branch exerts counter-regulatory, anti-inflammatory effects through the cholinergic anti-inflammatory pathway, making it a crucial yet often suppressed component of OA pathogenesis [39, 40].

In animal models, vagotomy accelerates OA development, with vagotomised rats showing thinner articular cartilage, enhanced subchondral bone remodelling, and increased matrix metalloproteinase activity compared with intact controls [41]. In OA patients, a disrupted sympatho-vagal balance is observed, characterized by increased sympathetic dominance and vagal withdrawal [36, 38–40]. This imbalance, combined with chronic inflammation, contributes to an elevated cardiovascular risk [6, 42]. Additionally, both cardiac autonomic regulation and the nociceptive response to acute experimental stress [39] or exercise [40] are impaired in OA patients. The heightened stress and pain levels commonly observed in these individuals create a vicious cycle where each component exacerbates the other, ultimately worsening disease severity [42]. In OA patients, beta-blocker use has been associated with reduced OA-related pain, further reinforcing the involvement of the sympathetic nervous system in the ANS-pain-inflammation interplay [36].

### Fibromyalgia

FM is a condition characterized by chronic widespread musculoskeletal pain [43], where pain plays a central role in the ANS-inflammation axis. The sympathetic nervous system can induce, facilitate, or amplify chronic pain through several mechanisms [44]. These putative mechanisms include increased responsiveness of injured sensory nerves to catecholamines, upregulation of α−1 adrenoreceptors on primary afferent nociceptors and hyperalgesic skin, central sensitization of Aβ mechanoreceptors, enhanced discharge and sympathetic sprouting in dorsal root ganglia, and impaired pain modulation [43, 44]. Interestingly, FM patients exhibit a blunted heart rate and blood pressure response to orthostatic stress compared to healthy controls [45], highlighting an abnormal balance in ANS. Moreover, in patients with fibromyalgia, the magnitude of autonomic adjustments to postural changes is inversely correlated with clinical pain severity, and aortic stiffening has also been reported, partly due to autonomic dysfunction [45, 46]. These findings suggest reduced autonomic flexibility and adaptability to stressful situations in FM.

## Immune dysfunction and anti-inflammatory vagus response

From a mechanistic standpoint, autonomic dysfunction and inflammation maintain a dynamic and bidirectional relationship [6, 47]. Experimental studies have shown that microinfusion of pro-inflammatory cytokines such as TNF-α, IL-6, and IL-1β into cardiovascular nuclei of the hypothalamus and brainstem augments sympathetic activity, suppresses parasympathetic tone, and reduces baroreflex sensitivity [48, 49]. Conversely, autonomic imbalance itself can amplify inflammatory signalling, as evidenced by the discovery of a neural feedback mechanism known as the “inflammatory reflex.”

Briefly, the central nervous system detects peripheral inflammation through afferent vagus nerve signalling mediated by circulating pro-inflammatory cytokines (and endotoxins). These signals modulate various activation pathways, including the nucleus of the solitary tract, triggering two distinct mechanisms of immune response: the cholinergic anti-inflammatory pathway (CAP) and the hypothalamic–pituitary–adrenal axis, to mitigate inflammation. The efferent vagus nerve pathway, in turn, is activated via the α7 subunit of nicotinic acetylcholine receptors (α7nAChRs) in macrophages, leading to reduced pro-inflammatory cytokine production through the mechanism of the CAP [47]. Through the efferent limb of the CAP, acetylcholine released by the vagus nerve binds to α7 nicotinic acetylcholine receptors (α7nAChRs) on macrophages, suppressing TNF-α, IL-1β, and IL-6 synthesis and thereby attenuating local and systemic inflammation.

Activation of α7nAChRs in macrophages can occur locally within inflamed joints or the spleen [47]. In the case of the hypothalamic–pituitary–adrenal axis [50], neurons in the hypothalamus activate the release of adrenocorticotropic hormone by the hypophysis, stimulating the release of glucocorticoids by the adrenal glands to decrease peripheral inflammation. This understanding of the inflammatory reflex has paved the way for novel therapeutic strategies, such as central and peripheral vagus nerve stimulation. Anti-inflammatory effects [51] and the balancing of immune responses have been reported, particularly through stimulation of the afferent pathways of the vagus nerve [52]. In patients with rheumatoid arthritis, vagus nerve stimulation has been shown to significantly inhibit the production of TNF-α and IL-6, reducing disease severity, even in cases resistant to pharmacological therapy [53].

## Gut-brain axis

Another important aspect is the gut-brain axis, which is central in modulating systemic inflammation, stress responses, and chronic pain. It has been demonstrated that vagal nerve function correlates with gut microbiome composition and is inversely associated with inflammatory markers [54, 55]. In addition, modulating the gut microbiome with probiotics could enhance vagus nerve function in participants with depression. Increasing evidence links chronic pain to intestinal barrier dysfunction, particularly through alterations in tight junction integrity [56]. Meta-analyses have identified higher rates of depression and elevated circulating biomarkers of gut barrier damage (e.g., zonulin, lipopolysaccharide-binding protein [LBP]) in individuals with impaired intestinal integrity [57]. Importantly, when one barrier system, such as the intestinal, blood–brain, or epithelial barrier, becomes compromised, others frequently follow, resulting in a state of low-grade systemic inflammation [56]. This “multi-barrier dysfunction” can manifest clinically as fatigue, pain sensitization, and depressive-like sickness behaviour, often corresponding to modest elevations in circulating C-reactive protein (3–5 mg/L) [58]. This multi-barrier dysfunction reinforces the inflammatory loop and may exacerbate symptoms in rheumatologic and mood disorders, especially when vagal activity may be insufficient to sustain the anti-inflammatory response [58].

Vagus nerve activation has emerged as a promising mechanism to counteract this process, promoting anti-inflammatory signalling and restoring barrier integrity through cholinergic pathways [59]. Rehabilitation stays, especially those incorporating environmental therapies, have been shown to produce significant de-stressing and restorative effects, possibly via enhanced vagus function [60]. However, the specific effects of radon balneotherapy on gut microbiome composition and epithelial barrier function remain poorly characterized. Preliminary findings from animal studies indicate that low-dose radon inhalation or immersion may modulate intestinal microbial communities in a dose-dependent manner, possibly through redox and immune-mediated mechanisms [61]. Similarly, serial mild hyperthermia interventions, common in radon spa settings, have been reported to alter gut microbial diversity and metabolic profiles, potentially contributing to systemic homeostatic adaptations [62]. These observations underscore the need for further research into the interplay between radon therapy, vagus nerve stimulation through activation of the gut-brain axis, and barrier integrity in chronic inflammatory diseases.

## Radon balneotherapy: putative mechanisms of action

Balneotherapy, a therapeutic approach involving the medical use of spa pools and mineral baths, is widely used to treat various chronic diseases, with heat being a key element in many protocols [63]. Among different balneotherapy techniques, the medicinal application of radioactive radon, known as radon baths, is an integrative approach for the treatment and rehabilitation of patients with rheumatologic conditions [11, 12, 64, 65]. Radon baths have been shown to reduce pain and rheumatologic symptoms, whilst providing other health benefits in clinical trials [11, 66, 67]. However, the underlying mechanisms responsible for these effects have yet to be completely elucidated. Moreover, the effective contribution of common co-determinants such as heat and NAI needs to be acknowledged and carefully considered. Nevertheless, it is plausible that the ANS plays a crucial role in mediating the therapeutic effects of radon baths. Previous studies have identified links between the ANS and key components of radon baths (such as heat exposure and NAI) and the role of the ANS in the pathophysiology of rheumatologic diseases has already been reasonably established [18, 19, 68]. This section will explore the physiological effects and mechanisms of action underlying this intervention.

From a radiobiological perspective, radon exposure delivers alpha-particle radiation in ultra-low doses, typically within the range of natural background exposure. This localized, low-linear energy transfer (LET) radiation can activate cellular defence mechanisms through a process known as radiation hormesis. The transient increase in reactive oxygen species (ROS) following alpha irradiation can stimulate adaptive upregulation of antioxidant enzymes such as superoxide dismutase, catalase, and glutathione peroxidase, thereby enhancing redox homeostasis rather than causing oxidative injury. Additionally, low-dose radiation has been shown to modulate DNA repair capacity and influence key signalling cascades, including NF-κB, Nrf2, and MAPK pathways, contributing to the attenuation of chronic inflammation and immune dysregulation [68, 69].

At the immunological level, radon-induced low-dose irradiation appears to shift cytokine profiles toward an anti-inflammatory phenotype, increasing IL-10 and TGF-β while downregulating pro-inflammatory mediators such as TNF-α, IL-1β, and IL-6 [70]. Experimental models further indicate that such exposure enhances macrophage polarization toward the M2 (regulatory) phenotype and supports the expansion of regulatory T cells (Tregs), mechanisms that may underlie the observed analgesic and disease-modifying effects in rheumatologic patients.

Passive heat exposure has been shown to modulate autonomic control of circulation, in both animal and human studies [71–75]. Whole-body passive heat stress is able to increase sympathetic activity while reducing vagal modulation to the heart. In contrast, localized heating has been shown to enhance cardiac vagal modulation, counteracting sympathetic dominance [72]. When applied repeatedly, heat therapy appears to induce adaptations in thermoregulatory mechanisms, ANS function, and hemodynamic control [72, 76].

Heat therapy via hot water immersion has been shown to modulate inflammatory responses [77], improve endothelial function, reduce arterial stiffness and blood pressure, and enhance cutaneous microvascular function through increased nitric oxide-dependent vasodilation [78, 79]. It also promotes the expression of heat shock proteins (HSPs), which play a crucial role in cellular protection and immune regulation [80, 81]. HSPs act as molecular chaperones that stabilize, refold, or degrade misfolded proteins under conditions of thermal or oxidative stress [82]. Their upregulation confers resilience against cellular injury, whereas impaired HSP expression can predispose tissues to apoptotic or necrotic damage [80–82]. Their upregulation is generally protective, and the absence of a heat shock response can be detrimental to cell survival [80–82]. In patients with rheumatoid arthritis, HSPs are upregulated at sites of inflammation, such as the synovial tissue [83, 84]. In this context, heat therapy can induce the expression of angiogenic mediators and HSP genes, particularly in skeletal muscle, contributing to tissue repair and homeostasis in healthy adults [85]. Importantly, experimental evidence shows that immunization with specific HSP isoforms, such as HSP60 and HSP70, promotes the expansion of regulatory T cells (Tregs) and attenuates pro-inflammatory immune responses, offering a plausible mechanism for heat-induced immune tolerance in rheumatoid arthritis [85].

Recent evidence indicates that HSPs also play a pivotal role in protecting skeletal muscle against oxidative stress, a major contributor to muscle atrophy in chronic inflammatory diseases. HSPs can downregulate pro-inflammatory cytokines such as TNF-α and IL-1β, both of which promote proteolysis. This is particularly relevant in rheumatoid arthritis, where sustained inflammation and elevated reactive oxygen species disrupt mitochondrial homeostasis and satellite cell function. Moreover, HSPs interact with the longevity-associated deacetylase Sirtuin 1 (SIRT1), which mitigates oxidative damage and preserves cellular energy metabolism [86]. The muscle-wasting phenotype characteristic of RA is further exacerbated by increased myostatin and IL-6 signaling via the JAK/STAT pathway; however, by stabilizing cytoskeletal structures and maintaining mitochondrial integrity, HSPs can counteract these catabolic drivers. Consequently, heat therapy, through the induction of HSPs, emerges as a promising non-pharmacological strategy to attenuate muscle loss and restore muscular resilience in RA [84].

Within this framework, the thermal component of radon balneotherapy may serve as both a physiological and radiobiological modulator of ANS and inflammatory activity. The combination of mild hyperthermia and ultra-low-dose alpha irradiation may synergistically enhance cellular defense pathways, promoting redox balance, mitochondrial stability, and stress protein activation. In this context, the hot water used during radon baths may act as a potential modulator of the ANS [71–73] and inflammation by activating HSPs [78–84]. Simultaneously, low-dose radon exposure may influence central autonomic regulation through indirect redox-mediated or neurohumoral mechanisms, potentially involving ROS-sensitive ion channels or nitric oxide signalling within autonomic nuclei. Among the proposed mechanisms, the interaction between NAI exposure and autonomic pathways in the brainstem has been suggested as a potential candidate [18]. Classic studies have reported reductions in heart rate and blood pressure in spontaneously hypertensive rats [84, 85], suggesting a suppression of the sympathetic nervous system and activation of the parasympathetic nervous system. Suzuki and colleagues [18] demonstrated that NAI can influence neuronal activity in autonomic circuits in the brainstem, potentially mediated by the vagus nerve. Specifically, exposure to NAI led to a marked decrease in c-Fos expression in the paraventricular nucleus and *locus coeruleus*, and a significant increase in c-Fos expression in the *nucleus ambiguus* and the *nucleus tractus solitarius*. This was accompanied by reductions in mean blood pressure and HR, as well as increased vagal-mediated HRV, namely in the high-frequency component. Notably, cervical vagotomy abolished these physiological and neuronal effects, supporting the role of the vagus nerve in mediating NAI responses [18, 87].

Although the influence of NAI on autonomic brain circuits has been described [18], the specific receptors or sensory pathways responsive to NAI have yet to be identified. It has been hypothesized that charged particles or reactive oxygen intermediates generated by NAIs could interact with airway epithelium or sensory neurons, triggering vagal afferent activation [88]. Afferent signals originating in the trachea are known to be conveyed to the central nervous system via the vagus nerve, terminating in the *nucleus tractus solitarius* [89]. It then projects to key autonomic nuclei, including the paraventricular nucleus, *locus coeruleus*, and *nucleus ambiguus*, which are involved in the modulation of ANS activity [90].

These findings are corroborated by a recent study that investigated the associations and molecular mechanisms linking forest NAI exposure to cardiac autonomic modulation in healthy adults [19]. These data suggest that NAI exposure may influence autonomic and inflammatory homeostasis through integrated redox and neuroimmune pathways, paralleling some of the effects observed with low-dose radon exposure. Higher NAI concentrations were associated with enhanced vagal-mediated HRV. Moreover, NAI-related metabolic changes reflected reductions in systemic inflammation and oxidative stress [19]. These results underline the possible effects that NAI may have toward improving cardiac autonomic function and inflammatory status.

Based on both clinical and experimental studies, NAI may represent a key mechanistic link between the ANS and inflammation, and a possible contributor to the therapeutic effects of radon baths in rheumatologic diseases. Pragmatic trials are warranted to evaluate these effects on autonomic regulation and inflammatory processes in individuals with rheumatologic conditions. In addition, controlled laboratory studies using both animal models and human participants should investigate how isolated and combined exposures to NAI and thermal stimuli influence cardiac autonomic modulation and inflammatory responses. In the following section, current findings on the effects of radon baths in rheumatologic diseases will be reviewed and summarized.

Taken together, the combined effects of thermal stimulation, low-dose alpha radiation, and NAI exposure may act synergistically to induce a systemic adaptive response consistent with the principles of low-dose radiobiology and hormesis. Through the activation of redox-sensitive transcription factors (e.g., Nrf2, NF-κB), upregulation of cytoprotective mediators such as HSPs and antioxidant enzymes, and modulation of autonomic and immune signalling networks, radon balneotherapy may enhance the organism’s resilience to inflammatory and oxidative stressors [91]. It is essential to note, however, that it is also possible that heat and NAIs may be mediating the effects of radon, and that radon may not confer these effects when administered in isolation. The effects of NAIs [92] and heat therapy [93] have been well-established and thus their combined effect on these adaptative responses cannot be ruled out. Nevertheless, this adaptive response, spanning molecular, cellular, and neurophysiological levels, offers a mechanistic framework for understanding some of the health benefits observed in clinical and experimental studies of radon spa therapy.

## Clinical evidence: effects of radon balneotherapy on rheumatologic diseases

The therapeutic application of radon-rich thermal waters has deep historical roots, with early uses dating back over two millennia in locations such as the Greek island of Ikaria [94]. The modern scientific understanding of this practice emerged in the early twentieth century, following the discovery of radioactive elements and gases by Marie and Pierre Curie. By the 1930 s, radon therapy had attained official medical recognition in several European countries and the former USSR [15, 16]. Treatment protocols typically involved either immersion in radon-enriched thermal waters or controlled inhalation of radon gas, administered under medical supervision to ensure dose safety and therapeutic efficacy. Since the 1980 s, clinical trials, including randomized double-blind studies, have investigated the efficacy of these interventions, particularly in alleviating pain and enhancing physical function in patients with rheumatic disorders [95]. This section explores the current clinical evidence regarding the effects of radon balneotherapy in managing rheumatologic diseases.

Radon balneotherapy has been studied in the context of rheumatic diseases, particularly for its analgesic, anti-inflammatory, and homeostatic-modulatory effects mediated by low-dose alpha radiation. Randomized controlled trials and meta-analyses have also provided support for its efficacy. A systematic review and meta-analysis by Falkenbach et al. [11] assessed five RCTs comprising 338 patients. Cumulative radon exposure, even at ultra-low doses, was associated with prolonged analgesic effects that extended beyond the immediate treatment period, consistent with an adaptive radiobiological response. Radon therapy showed superior pain reduction at 3- and 6- months following treatment, despite short-term pain relief not differing between intervention and control groups immediately post-therapy.

The IMuRa trial, a large multicentre randomized trial involving 681 patients, showed improvements in pain scores and a reduction in analgesic consumption for up to 9 months after receiving radon spa therapy [66]. Similarly, a study by Franke et al. [96] on patients with rheumatoid arthritis found that those who received radon-carbon dioxide baths had sustained reductions in pain and improved functional scores at 6 months post-treatment, whereas the control group returned to baseline levels. This persistence of benefit supports the hypothesis that low-dose alpha irradiation may induce long-lasting biological adaptations, potentially through modulation of redox-sensitive transcription factors (e.g., Nrf2, NF-κB) and neuroimmune signalling cascades that regulate inflammation and pain perception [94–96]. However, the effect of carbon dioxide or a synergistic interaction between radon and CO₂ exposure in the experiment cannot be ruled out.

On the other hand, a randomized, placebo-controlled, cross-over study involving 116 patients with musculoskeletal pain (RAD-ON02 trial) reported that the effects at 3- and 6-month post-therapy were comparable to the control group. Nevertheless, there were greater reductions in pain intensity and morning stiffness at 4 weeks compared to warm water baths alone, suggesting a faster onset of symptom relief using a combination of thermal and radiobiological stimuli [17, 97].

Van Tubergen and colleagues [98] compared radon exposure to sauna bathing and a control condition in patients with ankylosing spondylitis. The radon cave intervention involved 60-min sessions three times per week for 3 weeks (38–41.5 °C, 70–98% humidity, delivering 0.536 working level month). Of note, spa–exercise therapy improves functional ability and reduces pain in addition to drug treatment and weekly group physical therapy alone in patients with AS. A notable limitation, however, confounds the results: the intervention groups performed 1 h of exercise 5 days per week, whereas the control group exercised only once weekly. This difference in exercise frequency biases any attribution of effects solely to radon exposure.

From a mechanistic standpoint, inflammatory processes are a hallmark of rheumatologic diseases, and fascial connective tissue, rich in nociceptive and autonomic nerve endings, plays a central role in mediating pain. Painful contractions are often driven by fibroblast and myofibroblast activation under inflammatory conditions, which increases collagen deposition in the extracellular matrix. This fibrotic remodelling, coupled with oxidative stress, may sensitize mechanosensory and pain receptors, leading to chronic pain and reduced tissue elasticity [99].

Thermal stimulation, such as that induced by radon baths, enhances local metabolism and mitochondrial activity, establishing a positive feedback loop that supports cellular repair and energy homeostasis. The benefits of temperature fluctuation include fascial stiffness regulation, induction of anti-inflammatory genes (particularly SIRT1), and the facilitation of humoral and redox balance within the extracellular matrix. Radon balneotherapy may modulate these processes by restoring thermogenic function and promoting fascial relaxation through combined thermal and radiobiological effects, though this remains to be experimentally confirmed. Intermittent cold exposure might further enhance these responses by stimulating autonomic oscillation and vagal activity, while lack of temperature variability favours collagenous tissue rigidity. Indeed, SIRT1 has emerged as a key regulator of inflammatory processes in rheumatologic diseases [100]. Low-dose radon exposure, NAIs, or a combination of the two may activate SIRT1, contributing to anti-inflammatory and antifibrotic effects via suppression of myofibroblast activity and downregulation of the TGF-β pathway [101]. This provides a plausible molecular link between radon exposure, autonomic regulation, and improved musculoskeletal function.

It is essential to acknowledge that the therapeutic benefits observed in the clinical studies reviewed above cannot be unequivocally attributed to radon exposure alone. Several confounding factors are inherent to the multimodal spa environment, including concurrent exposure to heat, carbon dioxide, structured exercise or rehabilitation protocols, and NAIs. Only a limited number of studies have included control groups specifically designed to isolate radon-specific effects. For instance, van Tubergen and colleagues compared radon cave exposure to sauna bathing in patients with ankylosing spondylitis, but differential exercise frequencies between groups precluded definitive attribution of effects to radon alone [98]. Similarly, the RAD-ON02 trial compared radon spa therapy against warm water baths alone, reporting only short-term differences that did not persist at 3- or 6-month follow-ups [17, 97]. Franke and colleagues observed sustained clinical improvements following radon–carbon dioxide baths, yet the potential independent or synergistic contribution of carbon dioxide could not be ruled out [96]. Taken together, based on the current literature, radon-specific effects cannot be reliably separated from the broader multimodal spa context. Therefore, while radon balneotherapy may offer clinical benefits, these should be interpreted with caution, and future studies should employ rigorous controlled designs, including sham radon exposure under identical thermal and environmental conditions, to isolate the specific contribution of radon from its accompanying co-determinants.

## Risks, controversies, and regulatory restrictions

The biological effects and dose–response characteristics of chronic low-level radon exposure remain incompletely defined [102]. Radon, predominantly the isotope radon-222, is a noble gas generated by the decay of uranium, radium, and thorium [102, 103]. It is inhaled, equilibrates rapidly across the alveolar–capillary interface, and is largely exhaled unchanged [104]. Although radon gas itself is chemically inert, its short-lived radioactive progeny (notably ^218^Po and ^214^Po) deposit on the bronchial epithelium, where their α-particle emissions cause localized, high-linear-energy-transfer (LET) irradiation [105]. This results in the generation of reactive oxygen and nitrogen species, oxidative DNA lesions, and complex double-strand breaks that can initiate genomic instability, mutagenesis, and ultimately bronchopulmonary carcinogenesis [106].

However, these processes are modulated by cumulative dose, radiation quality, and individual susceptibility factors, including polymorphisms in DNA repair and antioxidant genes, rendering interindividual risk variable [107]. Intriguingly, at very low or intermittent doses, α-particle exposure can activate adaptive cellular pathways. Animal studies have shown that low-dose radon inhalation enhances antioxidant enzyme activity, upregulates stress-response genes, modulates cytokine signalling, and suppresses pro-inflammatory cascades, features consistent with a biphasic or hormetic dose–response [108]. Regardless, sustained exposure to large doses of radon still has a detrimental effect on health. This should be duly noted by researchers and clinicians seeking to use radon for therapeutic purposes.

Historically, high radon concentrations in mining environments were linked to elevated lung cancer incidence [109]. However, confounders such as male predominance, heavy smoking, and variable occupational exposure were often inadequately controlled [110], making direct extrapolation to residential settings problematic. From 1993 to 2007, the limits established by the International Commission on Radiological Protection (ICRP) were 600 Bq/m^3^ for homes and 1500 Bq/m^3^ for workplaces [111]; these were reduced in 2010 to 300 and 1000 Bq/m^3^, respectively, based on updated miner studies [112]. Current national limits are 300 Bq/m^3^ (EU), 148 Bq/m^3^ (USA), and 200 Bq/m^3^ (Canada, UK) [107]. The ICRP [111] calculates the annual effective dose using the following equation:

$$\mathrm{D}\mathrm{o}\mathrm{s}\mathrm{e}\;\mathrm{c}\mathrm{o}\mathrm{e}\mathrm{f}\mathrm{f}\mathrm{i}\mathrm{c}\mathrm{i}\mathrm{e}\mathrm{n}\mathrm{t} \times \mathrm{C}\mathrm{o}\mathrm{n}\mathrm{c}\mathrm{e}\mathrm{n}\mathrm{t}\mathrm{r}\mathrm{a}\mathrm{t}\mathrm{i}\mathrm{o}\mathrm{n} \times \mathrm{T}\mathrm{i}\mathrm{m}\mathrm{e}\; \mathrm{e}\mathrm{x}\mathrm{p}\mathrm{o}\mathrm{s}\mathrm{e}\mathrm{d}$$

Using a dose coefficient of 3 mSv per mJ·h·m^3^ and the equilibrium factor *F* = 0.4 between radon gas and its progeny for most indoor scenarios [113] corresponds to 6.9 × 10^−6^ mSv·Bq^−1^·h·m^3^, an indoor concentration of 200 Bq/m^3^, and 7000 h/year:

$$6.9 \times {10}^{-6} \mathrm{m}\mathrm{S}\mathrm{v}\cdot {\mathrm{B}\mathrm{q}}^{-1}\cdot \mathrm{h}\cdot {\mathrm{m}}^{3 }\times 200 \mathrm{B}\mathrm{q}/{\mathrm{m}}^{3} \times 7000 \mathrm{h}$$

yields approximately 9.66 mSv, which is what the ICRP annual threshold (10 mSv per year) is based on. For a typical 4-week radon therapy course, using the commission recommended dose coefficient of 6 mSv per mJ·h·m^3^ and the equilibrium factor *F* = 0.4, we have 1.4 × 10^−5^ mSv·Bq^−1^·h·m^3^. Multiplying this by a concentration level of 2080 Bq/m^3^ and an exposure of 1 h/day (4 weeks = 28 h):

$$1.4 \times {10}^{-5} \mathrm{m}\mathrm{S}\mathrm{v}\cdot {\mathrm{B}\mathrm{q}}^{-1}\cdot \mathrm{h}\cdot {\mathrm{m}}^{3} \times 2080 \mathrm{B}\mathrm{q}/{\mathrm{m}}^{3} \times 28 \mathrm{h}$$

This results in ~ 0.82 mSv [112, 114], which is under the annual dose threshold. However, it is important to note that the equilibrium factor of *F* = 0.4 is a conservative assumption recommended by the ICRP, which reflects the average conditions and provides a simplified method for estimating the effective dose. Concentration levels of caves may vary depending on ventilation, momentary weather, atmospheric pressure outside the caves, and different resorts/sites may have longer exposure times as part of their program. Nevertheless, users of radon therapy are advised to seek precise information regarding the locations they intend to visit and not conflate therapeutic-use exposures with chronic domestic risk [109].

**Fig. 1:**
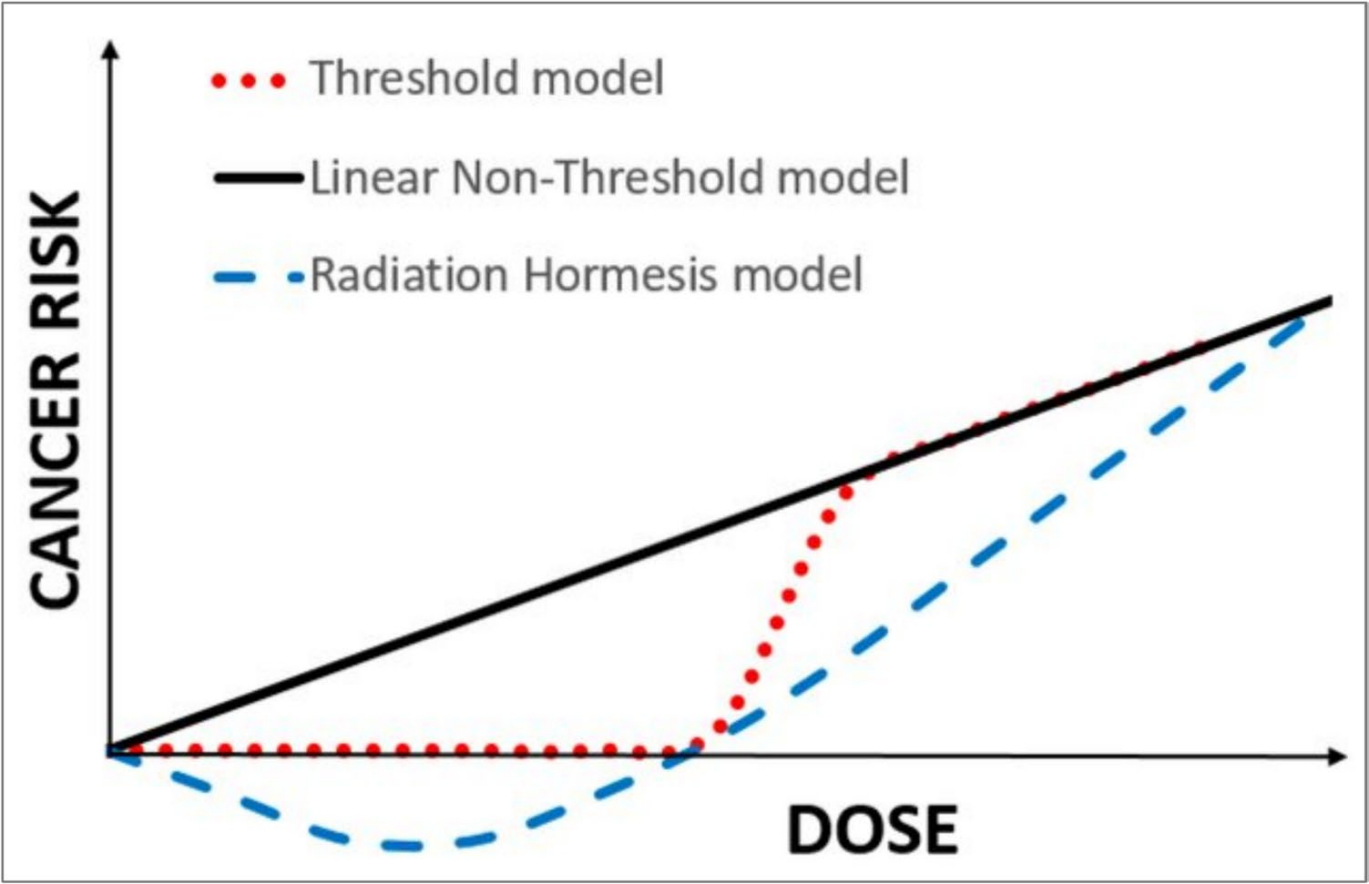
The three main dose–response models linking cancer risk to radiation dose. Adapted from Kabilan et al. [128]

Current radiological protection is anchored in the linear-non-threshold model (Fig. 1), which posits that cancer risk increases linearly with dose, with no safe threshold [107]. While suitable for high-dose extrapolation, the linear-non-threshold model may not accurately describe biological responses at low doses, where accumulating evidence supports threshold or hormetic behaviour [115]. Low-dose radiation can stimulate DNA repair fidelity, redox homeostasis, and immune surveillance, whereas high doses suppress these defences and cause cytotoxicity. Experimental work demonstrates that sub-toxic radon exposure enhances immune recognition and limits tumour progression [116]. Multi-omic analyses further reveal dose-specific gene networks, adaptive responses dominating at low doses, and damage-induced signalling at higher exposures [117, 118]. Notwithstanding, definitive conclusions still cannot be drawn, as prevailing evidence is conflicting on whether the linear-non-threshold model holds true at low doses [107, 119]. This is especially true given that the mechanism, and consequently the contribution through the “bystander” effect remains poorly understood [120]. Briefly, the “bystander” effect refers to the phenomenon that irradiated cells generate signals that induce a similar effect to unirradiated cells. Interestingly, this may not always be negative, as both adaptive [121], and protective responses [122] have been reported. However, the specifics are beyond the scope of this review and have been addressed elsewhere [123]. Despite evidence suggesting that biological systems exhibit nonlinear, homeostatic, and potentially beneficial adaptive responses to low-level ionizing radiation [124–126], the fundamental framework underlying the effects of ionizing radiation remains a point of contention [127].

In spite of some positive responses noted above, recent systematic reviews have confirmed associations between long-term residential radon exposure and lung cancer risk [129], though some evidence also suggests potential anti-inflammatory effects at high chronic exposure levels [130]. Consequently, many countries have enacted regulations to limit residential radon exposure. In the USA, homebuyers can request indoor radon measurements prior to purchase or rental, and testing companies require accreditation. In Ireland and the UK, detailed radon maps delineate concentration by administrative regions [117]. The EU has further developed a digital atlas reporting mean annual indoor radon concentrations over 10 × 10 km grids [131]. While European and US agencies share similar thresholds of concern, several European healthcare systems integrate radon-based therapies considered “alternative” in the USA [109]. For instance, in Poland, radon therapy is tightly regulated by the Ministries of Environmental Protection and Health and Social Welfare [132].

## Limitations

In spite of the current evidence, several methodological limitations persist. The populations being studied often have a pre-existing disease and are seeking a solution (i.e., self-selection bias and/or expectation bias). Many of the outcomes measured were based on self-reported questionnaires filled out by the participants, rather than more objectively quantifiable measures such as biochemical markers, tissue samples, or physiologically derived outcomes. Therefore, choice-supportive and confirmation biases, among others, cannot be ruled out. Moreover, studies investigating apparently healthy participants are still lacking. Experimental designs also require updating, as studies have now shown the efficacy of various long-term repeated heat therapy modalities such as hot water immersion and sauna bathing. Thus, it remains uncertain whether the therapeutic outcomes of radon balneotherapy arise primarily from thermal stimulation, radon exposure, NAIs, or a synergistic interaction between these factors. However, the thermal contribution is likely limited, as most radon baths are conducted at relatively mild temperatures, typically not exceeding 36 °C. At this temperature, head-out water immersion does not significantly elevate core body temperature, even after 60 min [133] or 120 min [134] and only induces a modest rise of approximately 0.5 °C after 3 h [135]. Moreover, clinical radon therapy sessions generally last 15–20 min, well below the duration required to elicit a physiologically meaningful hyperthermic response.

Therefore, the observed therapeutic effects are unlikely to be explained by passive heat accumulation alone and may instead reflect the biological actions of low-dose alpha radiation, such as redox modulation, anti-inflammatory signalling, and neuroimmune regulation, potentially augmented by mild thermal influences that improve peripheral circulation and radon uptake through the skin. Most of the research conducted thus far has also been based in health resorts, which often have their own unique set of protocols. Treatments are often multimodal [19] and include some form of physical therapy or rehabilitation, the use of massages or electrical stimulations, and also exercise. As the majority of the trials are run with an intent-to-treat, these adjunct activities may further alleviate patients’ symptoms. However, they also confound and/or mediate the results. In addition, there may also be inherent differences in radon concentration, mode of application (baths *vs.* inhalation), and treatment durations depending on the resorts, which limit result comparisons. These details need to be recorded and clearly reported in order to make it replicable for laboratory settings or other organizations looking to repeat the intervention.

## Future directions and research gaps

Although the effects of radon balneotherapy on mitigating rheumatologic symptoms have been documented, several critical questions remain unanswered, highlighting the need for a clearer understanding of its radiobiological basis, optimal dosing parameters, and systemic mechanisms of action. Further research is required to clarify how low-dose alpha radiation interacts with molecular and neural pathways to produce long-lasting therapeutic effects.

Recent hypotheses propose that radon balneotherapy may elicit systemic benefits not solely through its analgesic and anti-inflammatory actions but also via radiobiological modulation of ANS activity and redox homeostasis. Dysregulation of the ANS has been implicated in the pathophysiology of rheumatoid arthritis and related disorders, particularly in the context of reduced vagal tone and heightened sympathetic activity, both of which contribute to sustained inflammation and cardiovascular risk [6]. Preliminary findings indicate that radon exposure may enhance parasympathetic activity, possibly through redox-sensitive modulation of the vagus–spleen axis and other neuroimmune pathways linking the brain and immune system, although this requires validation in controlled mechanistic studies [101].

A major challenge in establishing clinical guidelines is the absence of well-characterized dose–response data and the limited understanding of individual radiosensitivity thresholds in low-dose radon therapy. The therapeutic response likely depends on multiple interacting variables, including age, sex, disease phenotype, inflammatory burden, genetic polymorphisms affecting redox enzymes, and baseline autonomic function. Consequently, standardizing exposure parameters such as concentration, duration, and frequency remains difficult [11]. The therapeutic outcome appears to be influenced by individual characteristics such as age, sex, disease type and severity, and autonomic responsiveness. As a result, standardizing exposure parameters remains a challenge [11]. Future studies should aim to isolate radon from adjunct and multimodal interventions (e.g., heat therapy, physiotherapy, massages) to quantify its effects more accurately. Patients should also be stratified based on pre-intervention health status, disease state, and/or biological markers to identify differential responders to optimize therapeutic regimens.

Given the elevated cardiovascular risk in patients with chronic inflammatory conditions, the radiobiological and neurophysiological potential of radon balneotherapy to modulate cardiovascular autonomic regulation warrants closer examination. Evidence from other environmental interventions suggests that therapies enhancing vagal tone and reducing sympathetic dominance can exert cardioprotective effects by dampening systemic inflammation, improving endothelial function, and stabilizing autonomic outflow [19]. Preliminary data indicate that low-dose alpha irradiation, as delivered during radon therapy, may similarly influence redox-sensitive autonomic pathways and vascular homeostasis through mild activation of the parasympathetic (cholinergic anti-inflammatory) reflex. However, radon balneotherapy has yet to be fully explored within this context. Longitudinal studies measuring HRV, baroreflex sensitivity, skin and muscle sympathetic nervous activity, and other biomarkers of cardiovascular autonomic control could provide important insights into its systemic benefits [136].

Longitudinal studies incorporating objective markers of autonomic and vascular regulation, such as HRV, baroreflex sensitivity, muscle and skin sympathetic nerve activity, endothelial-derived nitric oxide bioavailability, and inflammatory cytokine levels, are needed to clarify the systemic cardiometabolic benefits of therapeutic radon exposure.

Comparative trials evaluating radon balneotherapy alongside other environmental therapies, such as forest bathing (*Shinrin-yoku*), NAI exposure, or whole-body hyperthermia, are notably scarce. Given the shared mechanisms proposed for these interventions (e.g., modulation of oxidative stress, autonomic balance, and systemic inflammation), further studies could elucidate whether radon balneotherapy provides similar or synergistic effects in relation to these other non-pharmacological approaches.

To move radon balneotherapy from restricted practice to integrative clinical application, large-scale, multicentre, randomized controlled trials are needed. The majority of the current evidence is based on single-centre setups with limited generalizability and heterogeneous methodologies. Equally important is the development of international consensus regarding dosing protocols, therapeutic windows, and long-term safety. In particular, the appropriate risk documentation practices and complete transparent disclosure of information in accordance with up-to-date international guidelines [ICRP, United Nations Scientific Committee on the Effects of Atomic Radiation (UNSCEAR), WHO, etc.] need to be established. Additionally, research participants and patients alike need to be fully informed of the theoretical basis (e.g., hormetic vs linear no threshold model) behind the intervention. Existing data suggest that controlled low-dose exposure is generally well-tolerated, but comprehensive risk–benefit analyses remain incomplete and inconclusive [12].

Therefore, while radon balneotherapy shows promise as a supportive intervention for rheumatic diseases, its underlying radiobiological mechanisms, long-term systemic effects, and dose-dependent safety profiles remain insufficiently characterized. Bridging these knowledge gaps is essential not only to clarify its therapeutic potential but also to address public concerns and scientific debate surrounding low-dose radiation exposure. Comprehensive mechanistic and longitudinal studies will be critical to inform evidence-based safety standards, optimize exposure protocols, and guide policy frameworks for clinical implementation. Ultimately, a clearer mechanistic understanding will enable the development of scientifically grounded recommendations for the safe and effective utilization of radon therapy in modern rehabilitative and preventive medicine in the future.

## Conclusion

With its rich historical basis and accepted use in parts of Europe, radon balneotherapy could become a viable option amongst other non-pharmacological interventions for the management of rheumatologic diseases. A substantial body of evidence accumulated over decades lends credence to its use in relieving pain, particularly in rheumatologic diseases. Beyond symptomatic relief, contemporary radiobiological hypotheses propose that radon exposure may exert systemic effects through redox modulation and regulation of the ANS. The central role of the ANS in chronic inflammation, immune dysregulation, and cardiovascular comorbidity positions it as a plausible mechanistic target for low-dose radiation-based and environmental therapeutic approaches. In this context, adjunctive modalities such as heat therapy, NAIs and forest bathing, which exert similar effects on the autonomic nervous system and systemic inflammation, may provide complementary or synergistic effects when combined with radon therapy.

Nevertheless, substantial gaps remain in our understanding of the biological mechanisms, optimal dosing regimens, and long-term safety of radon exposure. There is an urgent need for rigorous mechanistic studies, beyond animal models, as well as large-scale randomized controlled trials to isolate and quantify radon’s specific biological and clinical effects. Doing so will ultimately allow us to fully elucidate the therapeutic potential of radon balneotherapy and its role within integrative healthcare frameworks.

## Supplementary Information

Below is the link to the electronic supplementary material.

ESM 1 (DOCX 23.9 KB)
