# Supplementary material for: Radon balneotherapy in rheumatologic diseases: potential mechanisms and implications for cardiovascular autonomic control and inflammation
Source: Clin Rheumatol. 2026 Jun 24;45(8):4775–90. doi: 10.1007/s10067-026-08202-y (PMC13486110; doi:10.1007/s10067-026-08202-y)
Supplement: Supplementary file 1 [file 10067_2026_8202_MOESM1_ESM.docx]

Table S1. Evidence table summarizing radon studies. This table provides an overview of clinical studies investigating the therapeutic effects of radon exposure (via baths, caves, or inhalation rooms) across various conditions, predominantly musculoskeletal and rheumatic diseases. The extracted data include study populations, intervention modalities, radon dosimetry, comparator groups, assessed outcomes, follow-up durations, and key methodological limitations.

| **Authors** | **Participant condition** | **Intervention components** | **Radon dose/concentration** | **Exposure time** | **Comparator** | **Key outcomes** | **Follow-up duration** | **Limitations (confounders)** |
| --- | --- | --- | --- | --- | --- | --- | --- | --- |
| Franke et al 2000 | rheumatoid arthritis, younger than 75 yrs of age | radon-carbon dioxide bath at 35 °C, 1.6 g CO2/L (250 L) | 1300 Bq/L (250 L) | 20 min, 15 sessions in 4 weeks | Carbon dioxide bath | pain (VAS), Keitel functional test (KFI), arthritis impact measurement scale (AIMS) | after treatment, 3 and 6 months after treatment | physiotherapy, massage, hydrogalvanic partial baths |
| Van Tubergen et al 2001 | ankylosing spondylitis | radon cave, 38 – 41.5 °C air temperature, 70 - 98% humidity | 0.536 working level month (WLM) | 60 min, 3x/week for 3 weeks | Multicentric; sauna bathing in a different location for second group, third group is control | Bath Ankylosing Spondylitis Functional Index (BASFI), pain (VAS), well-being (VAS), morning stiffness (in minutes) | 1 week after treatment, 13 weeks, 25 weeks, and 37 weeks after treatment | intervention groups did 1 hour exercise (5 days/week), control group only did exercise once a week |
| Mitsunobu et al 2003 | asthmatic adults, 23 – 79 years old | hot bathroom, 48 °C | 2080 Bq/m^3^ | 40 min, once a week, 5 sessions. | None (uncontrolled study) | pulmonary function test, antioxidant assay | after treatment | no control group or control condition, small sample size (n = 9) |
| Yamaoka et al 2004 | healthy males, 20 – 40 years old | hot bathroom 36 °C | 2080 Bq/m^3^ | 40 min, 5 sessions over 10 days | sauna 36 °C and 48 °C (two locations) both with radon concentration of 54 Bq/m^3^ | antioxidant levels (superoxide dismutase, catalase, lipid peroxide) | after treatment | short intervention length, participants were young healthy individuals |
| Franke et al 2007 | rheumatoid arthritis | radon-carbon dioxide baths at 35 °C, 1.3 g/CO2 | 1100 Bq/L | 20 min daily, 15 sessions | Carbon dioxide baths | self-assessed limitations in everyday life, private activities and occupational context (VAS) | after treatment, 3, 6, 9 and 12 months after treatment | leisure time sport and relaxation therapy not described or quantified |
| Franke et al 2013 | one of the following: back pain, osteoarthritis of hip or knee, rheumatoid arthritis, ankylosing spondylitis, spondylarthropathies, > 18 yrs old | radon baths at 36 – 38 °C or radon cave, 37 – 41.5 °C air temperature, 70 - 99% humidity | not reported | 20 min, 12 baths in 3 -4 weeks (every 2 – 3 days) | Multicentric; 7 locations, radon vs control | self-assessed pain (German Pain Society scale) | after treatment, 3, 6 and 9 months after treatment | different modalities and control conditions used at different centers, not all details of the 7 centers were provided, complementary interventions not mentioned, all participants resided near the centers |
| Kuciel-Lewandowska et al 2018 | osteoarthritis, 45 – 65 yrs old | radon baths and radon inhalations at 37 °C | Bath: 303.1–441.5 Bq/L, inhalation alpha radiation: 184.4–450.0 MeV | 15 min bath or 15 min inhalation every other day for 15 sessions in 21 days | Control group with no radon bath | total antioxidant status | after 18 days of treatment | control group were employees of the spa, 30 - 45 min of kinesiotherapy, physiotherapy not clearly reported |
| Kojima et al 2019 | cancer patients (colon, uterine, lung, liver) | Radon room, Respiro-radon therapy | Room: 200000 Bq/m^3^  Respiro-radon: 2 – 6 MBq/m^3^ | 40 min, 2x daily, then 4x/week | None | tumor markers, CT and MRI images | not applicable (case series) | case series |
| Van der Zee-Neuen et al 2023 | rheumatic musculoskeletal disease | radon bath at 37 °C and/or radon cave at 37 – 41.5 °C air temperature, 70 - 99% humidity | Bath: 707.57 Bq/L  Cave: 44000 Bq/ m^3^ | Bath: 20 min, 10 sessions, Cave: 60 min, 11 sessions  Treatment duration: 18 days (avg) | None | pain at rest, pain in motion (11-point numeric rating scale) | after treatment, 3, 6 and 9 months after treatment | longitudinal study, multimodal treatment including physical exercise, massages, lymphatic drainage and mud therapy. Treatment combinations not standardized |
| Eckert et al 2024 | musculoskeletal and chronic degenerative disorders of the spine and joints, 18 – 75 yrs old | Radon bath in thermoneutral (34 °C) water | 1200 Bq/L | 20 minutes, 3x/week for 3 weeks | Warm water bath, randomized crossover with 6 month washout | osteoclasts, mature osteoclasts, bone resorbing activity, osteoclast differentiation, markers of cartilage damage | after treatment, 3 and 6 months after treatment | same project as Donaubauer et al 2024 |
| Donaubauer et al 2024 | musculoskeletal disorders of the spine and joints, ≥40 yrs old | Radon bath in thermoneutral (34 °C) water | 1200 Bq/L (estimated cumulative dose: 1.3 mGy) | 20 minutes, 3x/week for 3 weeks | Warm water bath, randomized crossover with 6 month washout | Immunophenotyping, quantification of inflammatory mediators in serum | after treatment, 3 and 6 months after treatment | Safety concerns; therapy-related adverse events |

Abbreviations: AIMS: Arthritis Impact Measurement Scale; BASFI: Bath Ankylosing Spondylitis Functional Index; Bq/L: Becquerels per liter (measure of radioactivity concentration in water); Bq/m³: Becquerels per cubic meter (measure of radioactivity concentration in air); CO₂: Carbon dioxide; CT: Computed Tomography; KFI: Keitel Functional Index; MBq/m³: Megabecquerels per cubic meter; mGy: Milligray (measure of absorbed radiation dose); MRI: Magnetic Resonance Imaging; Rn: Radon; VAS: Visual Analog Scale; WLM: Working Level Month (cumulative exposure to radon decay products).

Notes:

- Radon Dose/Concentration: Units vary depending on the application medium (e.g., Bq/L for aqueous baths, Bq/m³ or WLM for atmospheric exposure in caves/rooms).
- Comparators: "None" indicates uncontrolled or single-arm study designs. In some controlled trials (e.g., Yamaoka et al., 2004), the comparator group was exposed to a low-dose radon environment rather than a true zero-radon control.
- Kuciel-Lewandowska et al. (2018): Alpha radiation is reported in MeV as a measure of patient exposure in the treatment rooms; the authors explicitly state that absorbed dose was not calculated, as it depends on individual anatomical variables (body fat content, absorption surface) and radon loss during use.
- Eckert et al. 2024 & Donaubauer et al. 2024: Both publications report different outcome parameters (osteoclast activity vs. inflammatory mediators) derived from the same randomized crossover trial.
